# *In planta* Genome Editing in Commercial Wheat Varieties

**DOI:** 10.3389/fpls.2021.648841

**Published:** 2021-03-15

**Authors:** Yuelin Liu, Weifeng Luo, Qianyan Linghu, Fumitaka Abe, Hiroshi Hisano, Kazuhiro Sato, Yoko Kamiya, Kanako Kawaura, Kazumitsu Onishi, Masaki Endo, Seiichi Toki, Haruyasu Hamada, Yozo Nagira, Naoaki Taoka, Ryozo Imai

**Affiliations:** ^1^Division of Applied Genetics, Institute of Agrobiological Sciences, National Agriculture and Food Research Organization (NARO), Tsukuba, Japan; ^2^Division of Basic Research, Institute of Crop Science, National Agriculture and Food Research Organization (NARO), Tsukuba, Japan; ^3^Institute of Plant Science and Resources, Okayama University, Kurashiki, Japan; ^4^Kihara Institute for Biological Research, Yokohama City University, Yokohama, Japan; ^5^Department of Agro-Environmental Science, Obihiro University of Agriculture and Veterinary Medicine, Obihiro, Japan; ^6^Biotechnology Research Laboratories, Pharma and Supplemental Nutrition Solutions Vehicle, Kaneka Corporation, Takasago, Japan

**Keywords:** wheat, genome editing, bombardment, CRISPR/Cas9, shoot apical meristem, particle bombardment, seed dormancy

## Abstract

Limitations for the application of genome editing technologies on elite wheat (*Triticum aestivum* L.) varieties are mainly due to the dependency on *in vitro* culture and regeneration capabilities. Recently, we developed an *in planta* particle bombardment (iPB) method which has increased process efficiency since no culture steps are required to create stably genome-edited wheat plants. Here, we report the application of the iPB method to commercially relevant Japanese elite wheat varieties. The biolistic delivery of gold particles coated with plasmids expressing CRISPR/Cas9 components designed to target *TaQsd1* were bombarded into the embryos of imbibed seeds with their shoot apical meristem (SAM) exposed. Mutations in the target gene were subsequently analyzed within flag leaf tissue by using cleaved amplified polymorphic sequence (CAPS) analysis. A total of 9/358 (2.51%) of the bombarded plants (cv. “Haruyokoi,” spring type) carried mutant alleles in the tissue. Due to the chimeric nature of the T0 plants, only six of them were inherited to the next (T1) generation. Genotypic analysis of the T2 plants revealed a single triple-recessive homozygous mutant of the *TaQsd1* gene. Compared to wild type, the homozygous mutant exhibited a 7 days delay in the time required for 50% seed germination. The iPB method was also applied to two elite winter cultivars, “Yumechikara” and “Kitanokaori,” which resulted in successful genome editing at slightly lower efficiencies as compared to “Haruyokoi.” Taken together, this report demonstrates that the *in planta* genome editing method through SAM bombardment can be applicable to elite wheat varieties that are otherwise reluctant to callus culture.

## Introduction

Genome editing using clustered regularly interspaced short palindromic repeats (CRISPR) and CRISPR-associated protein9 (Cas9) nuclease is a recent development which can greatly accelerate breeding efforts with precise modifications in traits of interest (Miao et al., 2013) and has already been applied to a variety of crop plants (Miao et al., 2013; Shan et al., 2014; Svitashev et al., 2015). In most cases, a CRISPR/Cas9 expression cassette, together with a selectable marker gene, is delivered to the plant cells through *Agrobacterium tumefaciens-* or biolistic delivery-mediated transformation methodologies. However, these methods generally require callus culture and regeneration processes that are lengthy, costly and labor-intensive. Furthermore, these conventional culture-based transformation methods are only applicable to genotypes that are amenable for cell culture and regeneration, which significantly limits the application of genome editing to commercial varieties in major crops such as wheat (*Triticum aestivum* L.), maize (*Zea mays* L.), soybean (*Glycine max* L.). To overcome these issues, the *in planta* particle bombardment (iPB) method which utilizes shoot apical meristem (SAM) as a target tissue for transformation was developed in wheat (Hamada et al., 2018). Within the SAM, the sub-epidermal (L2) cells maintain the potential to develop into germ cells (McDaniel and Poethig, 1988; Furner and Pumfrey, 1992; Irish and Sussex, 1992). Therefore, heritable mutations can be introduced into the L2 cells to generate stably edited materials. The iPB method has been successfully applied to genome-editing in a model variety of wheat (Hamada et al., 2018). The expression of CRISPR/Cas9 genes within the SAM of imbibed seed embryos induces genome editing and the plants grown from the embryos inherit the edited sequence to the next generation. However, application of this method to divergent varieties, including elite commercial cultivars, has not been tested until now.

Seed dormancy is one of the most important traits which affects the production of cereal crops. Specifically, pre-harvest sprouting (seed germination on the spike) leads to a serious decrease in yield and grain quality. Barley *Qsd1* (*quantitative trait locus on seed dormancy 1*) is one of the most effective QTL loci which was identified in a cross between European and Japanese cultivars (Hori et al., 2007). Subsequent map-based cloning efforts revealed that the *Qsd1* gene encodes a putative alanine aminotransferase (AlaAT) enzyme involved in nitrogen assimilation, carbon metabolism and protein synthesis through its reversible transfer of an amino group from glutamate to pyruvate, to form oxaloacetate and alanine (Miyashita et al., 2007). Experiments with knock-down transgenic plants indicated that the deficiency of *Qsd1* function may result in increased seed dormancy (Sato et al., 2016). An orthologous gene of the barley *Qsd1* was identified in common wheat (Onishi et al., 2017) and knocked-out *via Agrobacterium*-mediated transformation with a CRISPR/Cas9 gene cassette (Abe et al., 2019). In relative comparison to wild-type, a clear increase in seed dormancy was observed in a *qsd1* triple-recessive homozygous mutant (Abe et al., 2019). As a result, it is reasonable to consider that an introduction of the *Qsd1* knockout mutations into elite commercial cultivars using genome editing may have a significant impact on wheat breeding efforts. However, many elite commercial cultivars are recalcitrant to culture-based transformation methods and are therefore inaccessible to the benefits of tissue culture dependent genome editing strategies.

Here, we report on the genome editing of the *Qsd1* loci using the iPB method in genotypes that are recalcitrant to conventional transformation methods; demonstrating the successful introduction of a delayed germination phenotype into an elite Japanese wheat cultivar.

## Materials and Methods

### Preparation of Mature Embryos

Mature embryos were prepared according to previously described methodologies (Hamada et al., 2018; Imai et al., 2020). Briefly, mature seeds of wheat (*Triticum aestivum* L. cvs. “Haruyokoi,” “Yumechikara,” and “Kitanokaori”) were surface sterilized by 6% sodium hypochlorite with a commercial detergent for 20 min and rinsed several times with distilled water, and then germinated at 4°C for 2 days. SAMs of the mature seeds were exposed by removing the coleoptile and leaf primordia with an insulin pen needle 34 G (φ 0.2 mm; Terumo, Japan) under a stereomicroscope. The embryos separated from endosperms were placed upright on a petri dish containing Murashige and Skoog (MS) basal medium supplemented with maltose (30 g/L), 2-morpholinoethanesulfonic acid (MES) monohydrate (0.98 g/L, pH 5.8), plant preservative mixture (3%; Nacalai Tesque, Japan), and phytagel (7.0 g/L; Sigma Aldrich, United States). Thirty embryos were placed on the medium in each cycle for particle bombardment.

### Biolistic Delivery of Plasmid

Three plasmids expressing Cas9 [pE(R4-R3)ZmUbi_OsCas9_ ver3], GFP (pUba-GFP) (Hamada et al., 2018) and the guide RNA for TaQsd1 (pTaU6gRNA) (Kamiya et al., 2020), respectively, were mixed with 0.6 μm gold particles (Bio-Rad) and then bombarded into SAMs as previously described (Hamada et al., 2018). Biolistic bombardment was performed using a PDS1000/He particle bombardment device (Bio-Rad) with a target distance of 6.0 cm from the stopping screen to plate. The vacuum in the chamber was 27 inches of Hg and the helium pressure was 1,350 psi. The macrocarrier travel distance was 6 mm. Each petri dish was bombarded a total of four times.

### Plant Growth Condition

Mature embryos expressing GFP in SAMs were selected under a MZFLIII microscope (Leica) with a GFP filter 12 h after bombardment, and the embryos were transferred onto MS agar medium and cultured in growth chamber under a 14 h light/10 h dark photoperiod at 22°C for a total 3 weeks. Subsequently, healthy plants were transplanted to soil and cultivated in a growth room 20/13°C (day/night) under a 14 h light/10 h dark photoperiod. Vernalization was applied to the soil-grown winter cultivars under the condition of 4°C for a total of 4 weeks.

### PCR and Sequencing Analyses for Genome Edited Plants

Genomic DNA was isolated from leaf tissue as previously described (Hamada et al., 2018) and used as a template for PCR in a 15 μL reaction mixture using Ex *taq* polymerase (Takara, Japan). The thermal cycling program consisted of an initial denaturation at 94°C for 2 min followed by 30 cycles of amplification reactions; each consisting of 30 s denaturation at 94°C, 30 s annealing at 60°C and extension at 72°C for 30 s. The common primer and genome-specific primer sets were listed in Supplementary Table 2. The amplified PCR products were digested with the *Pst*I restriction enzyme and then analyzed by agarose gel electrophoresis. The undigested bands from the restriction enzyme reaction were then purified and cloned into the pGEM-T easy vector (Promega, United States), and then sequenced with a 3130xl Genetic analyzer (Applied Biosystems, United States). All sequences data analysis were processed by Geneious (Version 10.2.6).

### Phenotypic Analysis of *TaQsd1* Mutants

The levels of seed dormancy of the *TaQsd1* mutants were determined as previously reported (Abe et al., 2019). Briefly, six healthy seeds were directly sown in a pot with same amount of soil, and five pots for each WT and mutant line were used as replicates. The seeds from the primary and secondary spikes were harvested at 60 days after flowering, and 10 seeds from the center of a spike were placed in a Petri dish containing two sheets of filter papers moistened with 3 mL distilled water and then incubated in the dark at 20°C. The germination of the seeds was observed every day for a period of 30 days.

## Results

### Detection of CRISPR/Cas9-Mediated Genome Editing in T0 Plants

**FIGURE 1:**
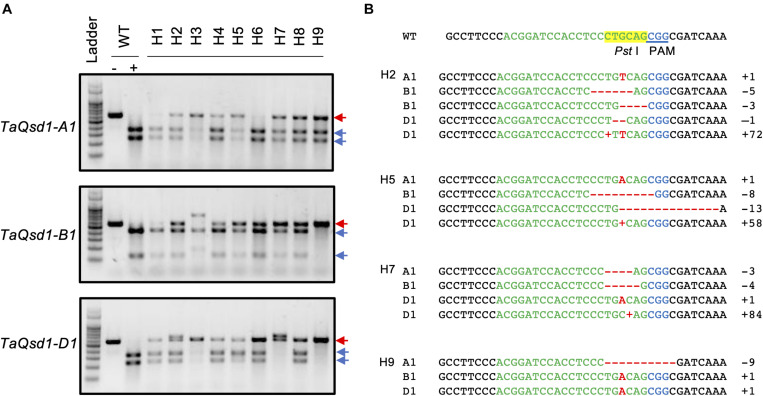
Genotyping of the mutant alleles of the *TaQsd1* locus in T0 plants. **(A)** Genomic DNA was isolated from the flag leaf of WT (cv. Haruyokoi) and the candidate T0 *taqsd1* plants and then subjected to a cleaved amplified polymorphic sequences (CAPS) assay. The PCR products were amplified by A, B, and D genome specific primer sets. −, undigested PCR products; +, *Pst*I digested PCR products. Red and blue arrows indicate undigested and digested bands after *Pst*I treatment, respectively. A 100 bp ladder was used as a size marker. **(B)** The genotypes of H2, H5, H7, and H9 as identified by sequencing. The green and blue characters indicate the gRNA and PAM sequences, respectively. The *Pst*I restriction site is highlighted in yellow. Red “+” indicates insertion of the 72, 58, or 84 bp vector sequence in H2-D, H5-D, or H7-d, respectively (Supplementary Figure 1) denotes the position of the long DNA fragment insertion. “+” indicates insertion of the 72, 58, and 84 bp of the vector sequence, respectively (Supplementary Figure 1).

To apply the iPB method to genome editing of the wheat *Qsd1* gene, a single guide RNA (sgRNA) targeting to all three of the homeologous genes (*TaQsd*-A1, -B1, and -D1) were designed according to the previous report (Abe et al., 2019). Three plasmid constructs carrying expression cassettes for *Staphylococcus pyogenes* (Sp) Cas9, the single guide (sg) RNA and a GFP reporter gene, respectively, were mixed with gold particles (Supplementary Figure 1). The DNA-coated gold particles were then bombarded at SAMs of imbibed embryos which were prepared according to previously described methods (Hamada et al., 2018; Imai et al., 2020). To conduct a large-scale screening for genome edited T0 plants, the bombarded embryos were grown first on an agar plate to establish root and shoot development and were then transferred to soil conditions. These plants were grown to maturity and flag leaves were collected and subjected to a cleaved amplified polymorphic (CAPS) analysis with the universal primers for the three homeologous *TaQsd1* genes which were designed to detect evidence of genome editing. A part of the CAPS analysis is shown in Supplementary Figure 2; three T0 plants (H1, H2, and H3) showed undigested bands after *Pst*I digestion, suggesting that mutations had occurred at the *Pst*I target site (Supplementary Figure 1). In total, we detected undigested bands in nine plants, accounting for 2.51% (9/358) of the bombarded embryos. The positive DNA samples were further CAPS-analyzed with three genome-specific primer pairs (Figure 1), which was then followed by Sanger sequencing for validation. All candidate T0 plants, with the exception of H1 and H6, showed mutant bands in three homeologous genes (Figure 1). Sequencing of some of the undigested bands revealed patterns of the mutation (Figure 1B). The mutations were detected in all three genomes and double and triple mutations were observed in some plants. The mutation pattern was divergent and included single base additions and short deletions. In addition, the insertion of vector-derived sequences was also detected (Figure 1B and Supplementary Figure 3). With the iPB method, it is possible that T0 plants developed from a bombarded SAM can be composed of independently-edited cells. Thus, multiple mutation patterns were detected in a genome of some individual T0 plants.

### Confirmation of Targeted Mutagenesis in the T1 Generation

A total of nine T0 plants (H1-H9) were selected and all of their T1 seeds were collected. Due to the chimeric nature of the T0 plants, it is possible that each seed from the same T0 plant can have a different genotype. Therefore, all the collected seeds were further analyzed by CAPS assay as summarized in Table 1, demonstrating that mutations were inherited to the T1 generation in six out of the nine T0 plants (Table 1).

**TABLE 1:** Summary of genotype analysis of T1 *taqsd1* plants.

Line	Spike	T1 seeds number	WT	Heterozygous*	Homozygous*
H1	main	32	32	0	0
	tiller	26	26	0	0
H2	main	14	5	1	8
	tiller	16	0	0	16
H3	main	32	32	0	0
	tiller	16	14	2	0
H4	main	32	32	0	0
	tiller	42	42	0	0
H5	main	14	0	0	14
	tiller	16	0	0	16
H6	main	16	16	0	0
	tiller	16	7	9	0
H7	main	16	16	0	0
	tiller	16	16	14	2
H8	main	7	7	0	0
H9	main	11	9	2	0

*The genotypes of T1 *taqsd1* plants were identified by CAPS analysis, in which the PCR were conducted with A, B, and D genome specific primer sets. Heterozygous: at least one copy of target gene was mutated, but not all genes were mutated; Homozygous: all six copy of target gene were mutated.

**FIGURE 2:**
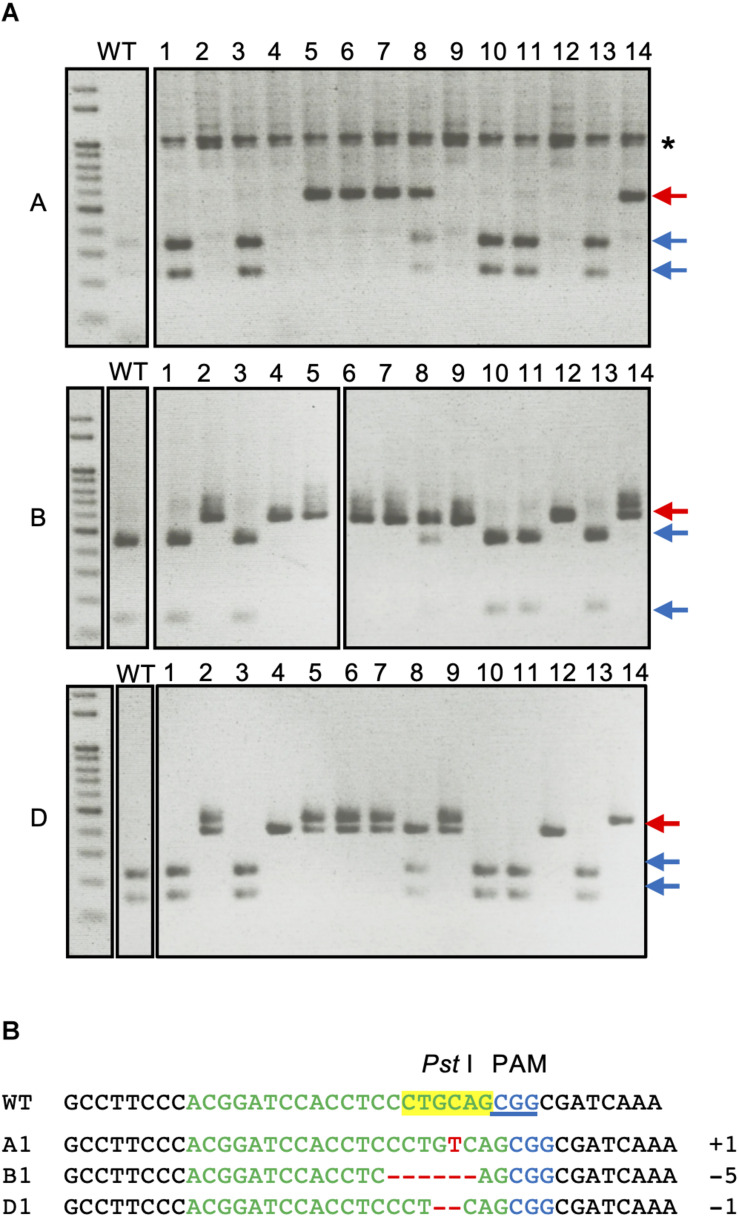
CAPS analysis of *TaQsd1* locus in first leaves of T1 wheat plants (from H2 plant). **(A)** Genomic DNA was isolated from the first leaves of WT (Haruyokoi cultivar) and representative T1 *TaQsd1* mutant plants and then subjected to CAPS assay. The PCR products were amplified by A, B, and D genome specific primer sets. Red and blue arrows indicate undigested and digested bands after *Pst*I treatment, respectively. The asterisk (*) denotes non-specific bands. **(B)** The sequence results of DNA extracted from the undigested bands of lane #8 (H2-8).

Cleaved amplified polymorphic sequence analysis of the first leaves of T1 plants from H2 plant with three genome-specific primer pairs were shown in Figure 2. The undigested bands were subsequently validated with Sanger sequencing and the details from the sequencing results of all the T1 plants are summarized in Supplementary Table 1. All of the genotypes detected in the T0 flag leaf of the H2 plant were identified and found to segregate in the T1 generation. Five T1 plants (H2-1, H2-3, H2-10, H2-11, and H2-13) were wild type for the *TaQsd1* gene. No *TaQsd1*-A genome products were amplified in four T1 plants (H2-2, H2-4, H2-9, and H2-12) with the specific primers utilized. One heterozygous T1 plant (H2-8) was successfully developed, whose genotype was confirmed as AaBbDd. The sequencing results confirmed that the H2-8 plant carried a single base T insertion in the A genome, a deletion of CCTGC in the B genome, as well as a G deletion in the D genome (Figure 2B). All mutations in the H2-8 plant resulted in frameshift mutations that introduced premature stop codons within the C-terminal region of the *TaQsd1* genes.

### Generation of Homozygous Edited Lines in the T2 Generation

**FIGURE 3:**
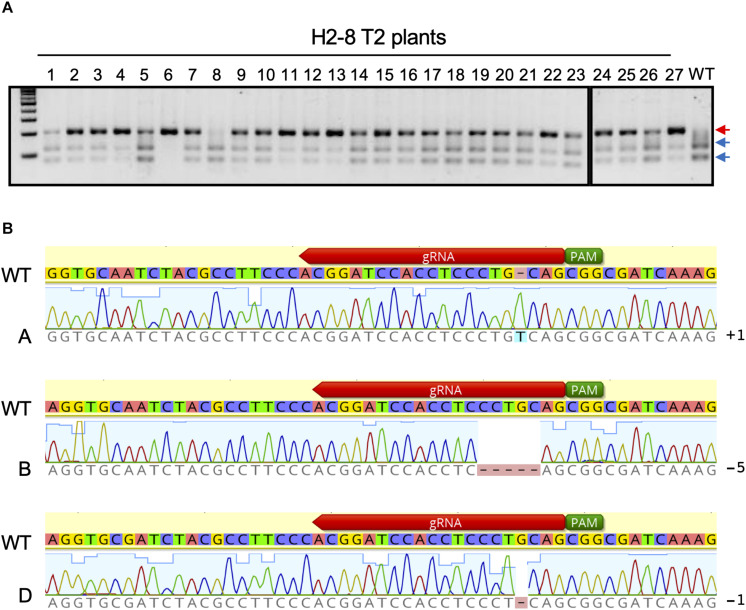
Isolation of a triple homozygous *taqsd1* plant from H2-8 plant progeny. **(A)** Genotyping of H2-8 plant progeny. The genomic fragments are amplified by A, B and D genome specific primer sets, and the PCR products are digested by *Pst*I restriction enzyme. Red and blue arrows indicate the sizes of undigested and digested bands after *Pst*I treatment, respectively. A 100 bp DNA ladder was used. **(B)** The sequences of the *TaQsd1* target region of H2-8-6.

The H2-8 line, which carries frameshift mutations in the A, B, and D homeologous genes was selected and grown for the identification of homozygous mutations in the T2 generation. All seeds of the H2-8 line were harvested and planted. First leaves from 27 T2 plants were then subjected to CAPS analysis with universal primer (Figure 3A). Of the 27 T2 plants that were analyzed, one (H2-8-6) showed no digested band, suggesting that the plant was a triple-recessive homozygous mutant. The remainder of the T2 plants showed digested band and were therefore considered as heterozygous for at least one genome. The genotype of the H2-8-6 plant was then validated by Sanger sequencing, and found to harbor an insertion of T, a deletion of CCTGC and a deletion of G in the A, B, and D genomes, respectively (Figure 3B). Since the mutations resulted in frame shifting at the C-terminal region (Supplementary Figure 4), the H2-8-6 mutant and its progeny was then used as a *TaQsd1* knockout line for subsequent phenotypic evaluations.

### A Homozygous Mutant Exhibited Prolonged Seed Dormancy

**FIGURE 4:**
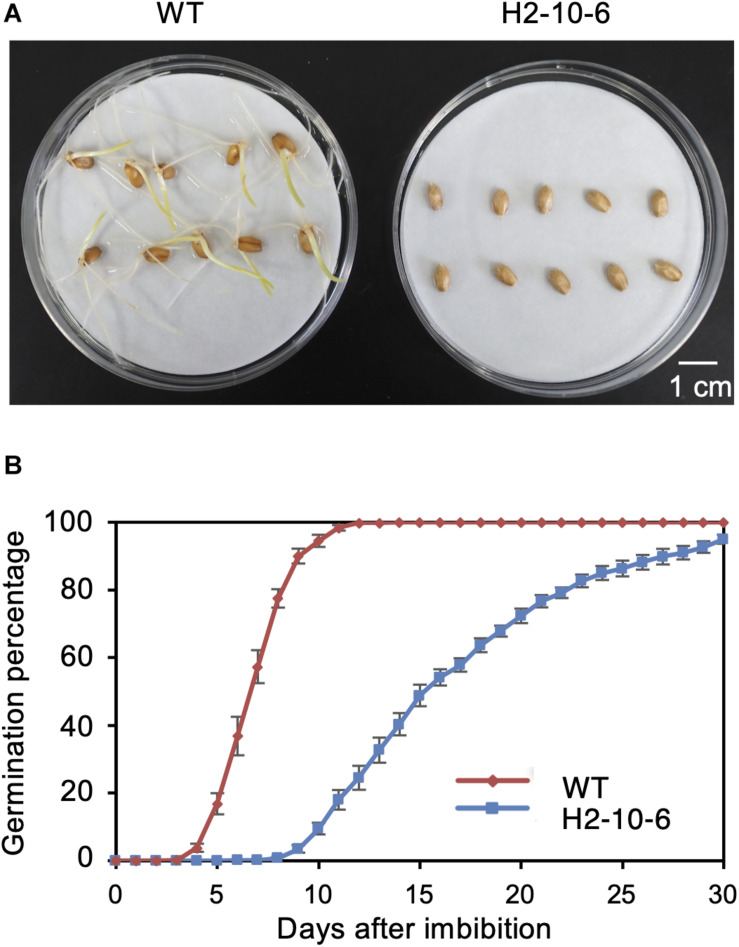
Effect of mutation of Haruyokoi *TaQsd1* in seed dormancy. **(A)** Germination of H2-8-6 mutant seeds on a Petri dish at 22°C in the dark. The photo was taken after 2-weeks from the beginning of the experiment. **(B)** The germination rate of WT and H2-8-6 in 30 days. Five biological replicates and 120 seeds in each replicate were used in this experiment.

Since the knockout of TaQSD1 function results in delayed seed germination (Abe et al., 2019), we measured the comparative germination rates between the H2-8-6 mutant and wild type plants. As shown in Figure 4, the H2-8-6 mutant exhibited a significantly retarded germination rate. The time to 50% seed germination in the homozygous mutant was delayed more than 7 days as compared to wild type material (Figure 4B). The overall time that was required for seeds to complete germination was more than 30 days for homozygous mutant, whereas this process was completed within 7–8 days for the wild type. Collectively, these data provide clear evidence which demonstrates that knocking out all three homeologous *TaQsd1* genes resulted in delayed germination in the commercially relevant “Haruyokoi” wheat cultivar.

### Applications to Other Cultivars

We also applied the same strategy to Japanese elite winter cultivars “Yumechikara” and “Kitanokaori.” The efficiency of the iPB-based genome editing was summarized in Table 2. In the case of “Yumechikara,” eight edited T0 plants were detected from a total of 884 bombarded embryos, accounting for a 0.9% mutation efficiency. Three of the eight T0 plants were subsequently confirmed to show successful inheritance of the mutations to the next generation, representing 0.3% of bombarded SAMs (Table 2). In the case of “Kitanokaori,” a single edited T0 plant was obtained from 183 bombarded embryos, accounting for a 0.5% mutation efficiency. Similar to what was observed in the other cultivars, this edited T0 plant also successfully passed the mutations to the T1 generation (Table 2). Collectively, these results confirmed that the iPB method can also be used for the genome editing of winter wheat cultivars.

**TABLE 2:** Summary of genome editing experiment on “Yumechikara” and “Kitanokaori” using the iPB method.

Cultivar	Number of bombarded SAMs	Number of plants with GFP signal expressed in SAM (%)*	Number of mutants in T0 progeny (%)**	Number of mutants in T1 progeny (%)**
Haruyokoi	358	298 (83.2)	9 (2.51)	6 (1.68)
Yumechikara	884	391 (44.2)	8 (0.9)	3 (0.3)
Kitanokaori	183	81 (44.3)	1 (0.5)	1 (0.5)

*The GFP fluorescence was detected 12 h after bombardment and embryos whose SAM gave positive signals were selected. **Mutants were identified through CAPS and sequence analyses in the flag leaf of T0 progeny or the first leaf of T1 progeny.

## Discussion

In this study, we reported the successful generation of knockout mutants of the *TaQsd1* genes in commercial wheat varieties, using genome editing *via* the iPB method. Importantly, we demonstrated that targeted heritable mutations resulted in a shift of an important trait in a commercially relevant cultivar. Specifically, the triple homozygous knockout mutant of *TaQsd1* exhibited longer seed dormancy as compared with wild type.

The iPB method utilizes SAM-exposed embryos as a target tissue for bombardment and allows them to proceed with *in planta* transformation/genome-editing. Importantly, this procedure does not require time-consuming callus culture and selection steps, and is therefore applicable to commercial varieties that are otherwise recalcitrant to callus culture and regeneration. Additional merits to use the iPB method over conventional culture-based methods include the avoidance of problems associated with somatic variations that occur during cultures and the utilization of mature seeds as a starting material, which skips the preparation of immature embryos.

When the iPB method was applied to the spring cultivar “Haruyokoi,” inheritable genome editing was observed within six out of 358 bombarded T0 plants (Table 2). The observed editing ratio (1.68%) was comparable to that of the method using bombardment on wheat immature embryos as the explant source (Zhang et al., 2016). Furthermore, our results confirmed that the iPB method can also be applicable to winter cultivars (Table 2), although the winter cultivars showed a lower genome editing ratio (0.9% in “Yumechikara” and 0.3% in “Kitanokaori”) as compared with the spring cultivars (Table 2; Hamada et al., 2018). We noticed that the SAMs of the winter cultivars were smaller and more fragile than what was observed in the spring cultivars. It is plausible that the SAM tissue of the winter cultivars might have been injured during the exposing procedure and bombardment, which can be reflected by the lower survival rates of the winter cultivars after bombardment (Table 2). As a result, the final genome editing ratio after bombardment is lower in the winter cultivars compare to the spring cultivars (Table 2; Hamada et al., 2018).

When applying the iPB method to wheat, the first three leaves were removed to expose the SAM. Genome editing can occur randomly in the cells within the SAM. Thus, T0 plants are genetically chimeric and several patterns of mutations can be detected within a T0 plant (Figure 1B). The spatial localization of the edited cells within the SAM of the embryos determines where the mutation can finally be detected in the mature plants (McDaniel and Poethig, 1988), which explains why mutated seeds were obtained from tillers, and not from the main culm in some T0 plants (Table 1).

Patterns of the mutations which were detected in T0 plants were diverse, including a one base addition and a few base deletions at the target genome site (Figure 1B). In addition, we observed the insertion of a short vector sequence in some of the mutants (Figure 1B and Supplementary Figure 3). As previous reported, biolistic delivery of DNA can cause fragmentation and rearrangement of the transgenes at the sites of integration as well as DNA rearrangements within the genome (Svitashev et al., 2002; Liu et al., 2019). This possibility has to be taken into consideration when this method is applied.

Mutants with such vector sequences can be excluded by PCR in the following generation.

A detailed segregation analysis of the H2-T1 plant revealed a complex mechanism of inheritance of T0 mutations (Supplementary Table 1). The flag leaf of the main culm of the H2-T0 plant is composed of cells with a small variation of mutations. Only one mutation allele was detected in the A genome and two mutation alleles were detected in the B and D genomes. These data suggested that a chimeric distribution of mutated cells existed within the flag leaf tissue. L2 cells of the flag leaf are derived from not one but a small number of the L2 cells of the bombarded SAMs. All of the mutation patterns in each genome observed with the T0 flag leaf were subsequently found in the T1 generation (Supplementary Table 1), indicating that the genotypes of the flag leaf and inflorescence are highly correlated.

In summary, we generated genome-edited *taqsd1* plants in commercial wheat cultivars by applying the iPB method. Since successful in several cultivars of wheat, it is plausible that the iPB method can be applied to many other crop species that are otherwise hard-to-transform or genome-edit with conventional culture-based methods. We believe that the iPB method will accelerate molecular breeding studies in commercial wheat cultivars and will be broadly applicable to advance genome-engineering research in crops.

## Data Availability Statement

The original contributions presented in the study are included in the article/Supplementary Material, further inquiries can be directed to the corresponding author/s.

## Author Contributions

RI conceived and supervised the study. RI and YL designed the experiments. YL, QL, and WL conducted the experiments. RI, YL, and WL analyzed data. FA and HHa assisted the experiments. FA, HHi, KS, YK, KK, and KO provided sequence information and assisted in the gRNA design. ME and ST helped facilitate vector construction. YL, WL, and RI wrote the manuscript. All authors contributed to the article and approved the submitted version.

## Conflict of Interest

HHa, YN, and NT were employed by Kaneka Corporation. RI receives research support from Kaneka Corporation. The remaining authors declare that the research was conducted in the absence of any commercial or financial relationships that could be construed as a potential conflict of interest.
