# Supplementary material for: In planta Genome Editing in Commercial Wheat Varieties
Source: Front Plant Sci. 2021 Mar 15;12:648841. doi: 10.3389/fpls.2021.648841 (PMC8006942; doi:10.3389/fpls.2021.648841)
Supplement: Supplementary file 1 [file Image_1.pdf]

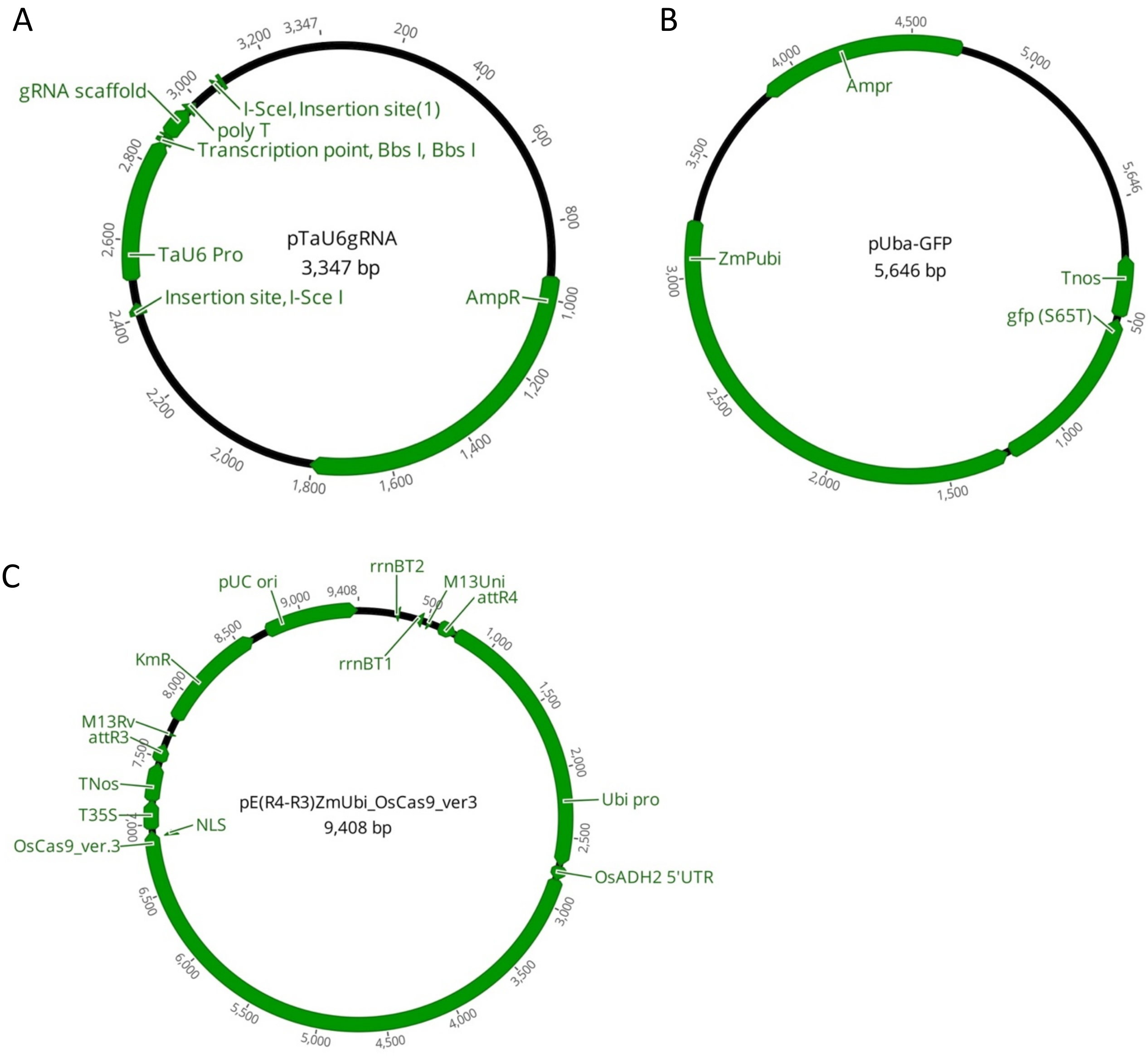

Supplementary Figure 1. The map of plasmid pTaU6gRNA (A), plasmid pUba-GFP (B) and plasmid pE(R4-R3)ZmUbi_OsCas9_ver3 (C).
