# Supplementary material for: In planta Genome Editing in Commercial Wheat Varieties
Source: Front Plant Sci. 2021 Mar 15;12:648841. doi: 10.3389/fpls.2021.648841 (PMC8006942; doi:10.3389/fpls.2021.648841)
Supplement: Supplementary file 2 [file Image_2.pdf]

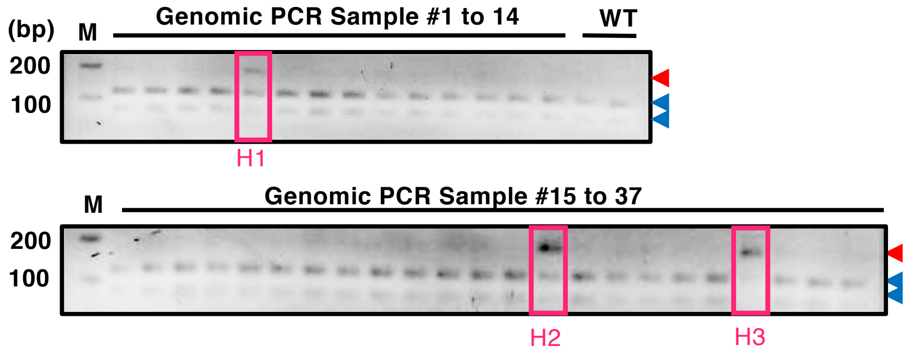

Supplementary Figure 2. A CAPS-based screening of TaQsd1 mutants using flag leaf tissue of bombarded T0 plants. A part of cleaved amplified polymorphic sequences (CAPS) assay data for T0 screening are shown. Genomic DNA was isolated from the flag leaf tissue of main culm of WT ('Haruyokoi') and bombarded T0 plants. A universal primer set was used to amplify all three genome clones. Red and blue arrows indicate undigested and digested bands after Pst I treatment, respectively. Samples showing positive signals are boxed.
