# Supplementary material for: In planta Genome Editing in Commercial Wheat Varieties
Source: Front Plant Sci. 2021 Mar 15;12:648841. doi: 10.3389/fpls.2021.648841 (PMC8006942; doi:10.3389/fpls.2021.648841)
Supplement: Supplementary file 3 [file Image_3.pdf]

A

	DNA sequence (5’-3’)
**+72**	AACGCAGCAGTTCCTCTTAGTTTAGTCCCACCTCGCCTGTCCAGCAGAGTTCTGACCGGTTTATAAACTCGC
**+58**	ATAGCTCACGCTGTAGGTATCTCAGTTCGGTGTAGGTCGTTCGCTCCAAGCTGGGCTG
**+84**	TATTTGTTTATTTTTCTAAATACATTCAAATATGTATCCGCTCATGAGACAATAACCCTGATAAATGCTTCAATAATATTGAGC

B

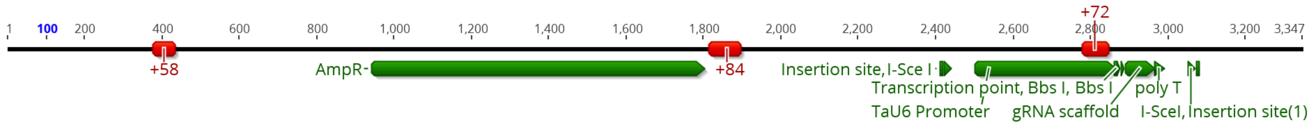

Supplementary Figure 3. DNA sequences of the inserted fragments (A) and their location within the plasmid pTaU6gRNA (B).
