# Supplementary material for: In planta Genome Editing in Commercial Wheat Varieties
Source: Front Plant Sci. 2021 Mar 15;12:648841. doi: 10.3389/fpls.2021.648841 (PMC8006942; doi:10.3389/fpls.2021.648841)
Supplement: Supplementary file 4 [file Image_4.pdf]

```
WT-A: MSYNKTASITAETINPKVKIFDYEPCGEIARHAERLEQEMEKSPGSRPFPEITYCNLGNPQALGQRPITFFREVLSLCDNPALLRR
H2-8_A: MSYNKTASITAETINPKVKIFDYEPCGEIARHAERLEQEMEKSPGSRPFPEITYCNLGNPQALGQRPITFFREVLSLCDNPALLRR

DETRMLFSPCAINRARKIIESMPGRNSGAYTNSQGIRSLREAVASGIAARDGFPSRPEDIFLTDGASSAINLSMQILIRSQEDGVL
DETRMLFSPCAINRARKIIESMPGRNSGAYTNSQGIRSLREAVASGIAARDGFPSRPEDIFLTDGASSAINLSMQILIRSQEDGVL

CPLPEYPLYSASIILHGGTMVPYNLSEDGDWGLEIFEVKRCLEEARIAGLTVRAMVIINPGNPTGQVLSITNQEEIVEFCRKEGLV
CPLPEYPLYSASIILHGGTMVPYNLSEDGDWGLEIFEVKRCLEEARIAGLTVRAMVIINPGNPTGQVLSITNQEEIVEFCRKEGLV

MLADEVYQDNVYVEDRKFHSFKKVARSLGYDENDISIVSFHSVSMGFSGECGRRGGYMEICGFGDDVMGEIRKVASVTLCPNIGGQ
MLADEVYQDNVYVEDRKFHSFKKVARSLGYDENDISIVSFHSVSMGFSGECGRRGGYMEICGFGDDVMGEIRKVASVTLCPNIGGQ

ILTSLAMDPPKLGDGCFENFMAEKEDIRLSLAKRAKTLSSAFSSLEGMTCNKVEGAIYAFPRIHLPAAAIKAAKAEGVSPDMFYAC
ILTSLAMDPPKLGDGCFENFMAEKEDIRLSLAKRAKTLSSAFSSLEGMTCNKVEGAIYAFPRIHLPVSGDQSRQGRGRVPRHVLRV

RLLDATGIAVVPGSGFHQVSGRNKATGTCHIRCTILPGEEKIKEMIPRLKEFHESFMNEFRDRS*
PPSRRHRDRRRPWLWIPPGVWAQQGHRDMSYPVHDPPGRGEDQGDDPAPQGVPRVLHERVPRPKLMCIFSYTSHRPVRALKAV*


WT-B: MSYNKTASITAETINPKVKIFDYEPCGEIARHAERLAQEMEKSPGSRPFPEITYCNLGNPQALGQRPITFFREVLSLCDNPALLHRM
H2-8_B: SYNKTASITAETINPKVKIFDYEPCGEIARHAERLAQEMEKSPGSRPFPEITYCNLGNPQALGQRPITFFREVLSLCDNPALLHR

DETRMLFSPCAINRARKIIESMPGRNSGAYTNSQGIRSLREAVANGIAARDGFPSRPEDIFLTDGASSAINLSMQILIRSQEDGIL
DETRMLFSPCAINRARKIIESMPGRNSGAYTNSQGIRSLREAVANGIAARDGFPSRPEDIFLTDGASSAINLSMQILIRSQEDGIL

CPLPEYPLYSASIILHGGTMVPYNLSEDGDWGLEIFEVKRCLEEARIAGLTVRAMVIINPGNPTGQVLSITNQEEIVEFCRKEGLV
CPLPEYPLYSASIILHGGTMVPYNLSEDGDWGLEIFEVKRCLEEARIAGLTVRAMVIINPGNPTGQVLSITNQEEIVEFCRKEGLV

MLADEVYQDNVYVEDKKFHSFKKVARSLGYDENDISIVSFHSVSMGFSGECGRRGGYMEICGFGDDVMGEIRKVASVTLCPNTSGQ
MLADEVYQDNVYVEDKKFHSFKKVARSLGYDENDISIVSFHSVSMGFSGECGRRGGYMEICGFGDDVMGEIRKVASVTLCPNTSGQ

ILTSLAMDPPKLGDGCFEDFMAEKEDIRLSLAKRAKTLASAFSSLEGMTCNKVEGAIYAFPRIHLPAAAIKAAKAEGMSPDMFYAC
ILTSLAMDPPKLGDGCFEDFMAEKEDIRLSLAKRAKTLASAFSSLEGMTCNKVEGAIYAFPRIHLSGDQSRQGRGHVSRHVLRVPP

RLLNATGIAVVPGSGFHQVSGRNKATGTWHIRCTILPGEDKIKAMIPRLKEFHESFMNEFRNRS*
SQRHRDRRCPWLWIPPGVWAQQGHRDMAYPVHDPPRRGQDQGDDPAPQGVPRVLHERVPQPKLMRTFTYTSHRPVRAPKV*


WT-D: MSYNKTASITAETINPKVKIFDYEPCGEIARHAERLEQEMEKSPGSRPFPEITYCNLGNPQALGQRPITFFREVLSLCDNPALLRRM
H2-8_D: SYNKTASITAETINPKVKIFDYEPCGEIARHAERLEQEMEKSPGSRPFPEITYCNLGNPQALGQRPITFFREVLSLCDNPALLRR

DETRMLFSPCAINRARKIIESMPGRNSGAYTNSQGIRSLREAVASGIAARDGFPSRPEDIFLTDGASSAINLSMQILIRSQEDGVL
DETRMLFSPCAINRARKIIESMPGRNSGAYTNSQGIRSLREAVASGIAARDGFPSRPEDIFLTDGASSAINLSMQILIRSQEDGVL

CPLPEYPLYSASIILHGGTMVPYNLSEDGDWGLEIFEVKRCLEEARIAGLTVRAMVIINPGNPTGQVLSVTNQEEIVEFCRKEGLV
CPLPEYPLYSASIILHGGTMVPYNLSEDGDWGLEIFEVKRCLEEARIAGLTVRAMVIINPGNPTGQVLSVTNQEEIVEFCRKEGLV

MLADEVYQDNVYVEDRKFHSFKKVARSLGYDENDISIVSFHSVSMGFSGECGRRGGYMEICGFGDDVMGEIRKVASVTLCPNIGGQ
MLADEVYQDNVYVEDRKFHSFKKVARSLGYDENDISIVSFHSVSMGFSGECGRRGGYMEICGFGDDVMGEIRKVASVTLCPNIGGQ

ILTSLAMDPPKLGDGCFENFMAEKEDIRLSLAKRAKTLAGAFSSLEGMTCNRVEGAIYAFPRIHLPAAAIKAAKAEGMSPDLFYAC
ILTSLAMDPPKLGDGCFENFMAEKEDIRLSLAKRAKTLAGAFSSLEGMTCNRVEGAIYAFPRIHLPQRRSKPPRPRACLQTCSTRA

RLLDATGIAVVPGSGFHQVSGRNKATGTWHIRCTILPGEDKIKVMIPRLKEFHESFMNEFRDRS*
AFSTPLGLPSSLALDSTRCLGETRPPGHGISGARSSPARTRSR*
```

Supplementary Figure 4. The alignment of TaQSD1sequences encoded by A, B, D genome of WT and H2-8 plants, respectively.

The upper is the wild type amino acid sequence and the lower is the genome-edited sequence of TaQSD1. The altered amino acids are indicated by red color.
