# Supplementary material for: In planta Genome Editing in Commercial Wheat Varieties
Source: Front Plant Sci. 2021 Mar 15;12:648841. doi: 10.3389/fpls.2021.648841 (PMC8006942; doi:10.3389/fpls.2021.648841)
Supplement: Supplementary file 5 [file Table_1.PDF]

Supplementary table 1. Summary of genotypes of T1 *taqsd1* plants (from H2 plant).

Generation	No.	A genome		B genome			D genome		
T0	H2	Ins. T	WT	Del. CAG	Del. CCTGC	WT	Del. G	Ins. 72bp	WT
T1	H2-1	-	WT	-	-	WT	-	-	WT
T1	H2-2	-	-	Del. CAG	Del. CCTGC	-	Del. G	Ins. 72bp	-
T1	H2-3	-	WT	-	-	WT	-	-	WT
T1	H2-4	-	-	-	Del. CCTGC	-	Del. G	-	-
T1	H2-5	Ins. T	-	Del. CAG	-	-	Del. G	Ins. 72bp	-
T1	H2-6	Ins. T	-	Del. CAG	-	-	Del. G	Ins. 72bp	-
T1	H2-7	Ins. T	-	-	Del. CCTGC	-	Del. G	Ins. 72bp	-
T1	H2-8	Ins. T	WT	-	Del. CCTGC	WT	Del. G	-	WT
T1	H2-9	-	-	Del. CAG	-	-	Del. G	Ins. 72bp	-
T1	H2-10	-	WT	-	-	WT	-	-	WT
T1	H2-11	-	WT	-	-	WT	-	-	WT
T1	H2-12	-	-	Del. CAG	-	-	Del. G	-	-
T1	H2-13	-	WT	-	-	WT	-	-	WT
T1	H2-14	Ins. T	-	Del. CAG	-	-	-	Ins. 72bp	-

The genotypes of T1 *taqsd1* plants (H2 line) were identified by sequencing. Ins.: insertion; Del.: deletion; - : not amplified by PCR; Ins. 72bp: Insertion of 72bp of TaU6 promoter (gRNA construct).
