# Supplementary material for: In planta Genome Editing in Commercial Wheat Varieties
Source: Front Plant Sci. 2021 Mar 15;12:648841. doi: 10.3389/fpls.2021.648841 (PMC8006942; doi:10.3389/fpls.2021.648841)
Supplement: Supplementary file 6 [file Table_2.PDF]

Supplementary Table 2. Primers used in this study.

Description	Sequence (5'-3')
TaQsd1Aspe_3779F	CACATTGTCAACAAGCACACCA
TaQsd1Aspe_4327R	GGAGCAAAATGAGTGAATCCGTA
TaQsd1Bspe_3864F	CTGGCCCTCATGTGGTCTTC
TaQsd1Bspe_4366R	GGGATCATCGCCTTGATCTTG
TaQsd1Dspe_3602F	CATACGCACTGCCTCCTTTTCA
TaQsd1Dspe_4124R	GTTTCGCCCAGACACCTTTGTT
TaQsd1-common F	CAGCCTGGAGGGAATGACC
TaQsd1-common R	ACCTGGTGGAATCCAGAGC
